# Robotic Postural Training With Epidural Stimulation for the Recovery of Upright Postural Control in Individuals With Motor Complete Spinal Cord Injury: A Pilot Study

**DOI:** 10.1089/neur.2024.0013

**Published:** 2024-03-15

**Authors:** Enrico Rejc, Collin Bowersock, Tanvi Pisolkar, Isirame Omofuma, Tatiana Luna, Moiz Khan, Victor Santamaria, Beatrice Ugiliweneza, Claudia A Angeli, Gail F Forrest, Joel Stein, Sunil Agrawal, Susan J Harkema

**Affiliations:** ^1^ Tim and Caroline Reynolds Center for Spinal Stimulation, Kessler Foundation, West Orange, New Jersey, USA.; ^2^ Department of Medicine, University of Udine, Udine, Italy.; ^3^ Kentucky Spinal Cord Injury Research Center, University of Louisville, Louisville, Kentucky, USA.; ^4^ Department of Mechanical Engineering, Northern Arizona University, Flagstaff, Arizona, USA.; ^5^ Department of Mechanical Engineering, Columbia University, New York, New York, USA.; ^6^ Department of Radiology at BWH, Harvard Medical School, Boston, Massachusetts, USA.; ^7^ Department of Physical Therapy, New York Medical College, Valhalla, New York, USA.; ^8^ Department of Neurological Surgery, University of Louisville, Louisville, Kentucky, USA.; ^9^ Department of Physical Medicine and Rehabilitation, Rutgers New Jersey Medical School, Newark, New Jersey, USA.; ^10^ Department of Rehabilitation and Regenerative Medicine, Columbia University, New York, New York, USA.

**Keywords:** epidural stimulation, postural control, spinal cord injury, standing, training

## Abstract

Activity-based training and lumbosacral spinal cord epidural stimulation (scES) have the potential to restore standing and walking with self-balance assistance after motor complete spinal cord injury (SCI). However, improvements in upright postural control have not previously been addressed in this population. Here, we implemented a novel robotic postural training with scES, performed with free hands, to restore upright postural control in individuals with chronic, cervical (*n* = 5) or high-thoracic (*n* = 1) motor complete SCI, who had previously undergone stand training with scES using a walker or a standing frame for self-balance assistance. Robotic postural training re-enabled and/or largely improved the participants' ability to control steady standing, self-initiated trunk movements and upper limb reaching movements while standing with free hands, receiving only external assistance for pelvic control. These improvements were associated with neuromuscular activation pattern adaptations above and below the lesion. These findings suggest that the human spinal cord below the level of injury can generate meaningful postural responses when its excitability is modulated by scES, and can learn to improve these responses. Upright postural control improvements can enhance functional motor recovery promoted by scES after severe SCI.

## Introduction

Motor complete, high-level spinal cord injury (SCI) disrupts the communication between supraspinal and spinal centers, leading to severe impairment of trunk control and to the inability to stand, walk, and move the lower limbs voluntarily. Postural control is a foundational ability to perform these motor tasks, and it is also drastically impaired after SCI.^1–4^ In the last decade, proof-of-principle studies showed that spinal cord epidural stimulation (scES) combined with activity-based training can promote remarkable recovery of standing, walking, and voluntary leg movements in individuals with chronic, clinically motor complete SCI.^5–10^ However, improvements in upright postural control have not been reported or directly tested yet. Importantly, self-assistance for postural control is regularly implemented by individuals with SCI during overground standing and walking with scES, where the participants place their hands on assistive devices such as a walker or standing frame. Retraining bipedal upright motor functions while concurrently providing mechanical self-stabilization by upper limbs results in compensatory postural control strategies, which can reduce or even suppress lower limb postural responses.^11–13^ Further, the required placement of upper limbs on assistive devices limits the potential for functional interaction with the environment.

Research on animal models observed that postural control deficits are related to the extent and location of SCI,^14^ highlighting the importance of supraspinal inputs for this aspect of motor control.^15–19^ However, studies in spinalized and/or decerebrated animal models,^20–23^ which rely entirely on the integration of limb-related somatosensory information to adjust posture,^23^ provided evidence that the mammalian spinal circuitry can retain the capability to regain partial postural control when scES is applied and postural training is practiced.^24,25^ Trunk postural control was also found importantly associated with stepping performance in rats receiving spinal cord stimulation.^26^ In ambulatory individuals with incomplete SCI, postural stand training with visual feedback showed the potential to improve postural control during stable and dynamic standing.^27^ We have also observed evidence that the human spinal circuitry below the level of a clinically motor complete SCI can interpret postural-related sensory information and generate lower limb postural responses when its excitability is properly modulated by scES parameters selected to facilitate standing (Stand-scES). We reported that body weight (BW) shifting controlled by upper limbs on a fixed handlebar during standing with Stand-scES can promote meaningful lower limb activation pattern modulation.^6^ More recently, we also assessed the effects of postural perturbations delivered at the trunk while individuals with motor complete SCI were standing with Stand-scES.^28^ Trunk perturbations elicited distinct lower limb postural responses, which were generally more frequent, larger in magnitude, and appropriately modulated when the participants' hands were free rather than placed on a fixed handlebar for self-balance assistance.^28^

These findings support the view that scES coupled with enhanced technology for postural training may be beneficial for exploiting the postural control potential that conceivably resides in the human spinal cord after a clinically motor complete SCI. In particular, a robotic upright stand trainer (RobUST) has been designed and validated for the control of human standing balance at ROAR Laboratory (Columbia University).^11,29^ The RobUST is a motorized cable-driven device that can provide assistance as needed and deliver controlled perturbation forces at the trunk and pelvis.

Here, we implemented ∼80 sessions of robotic postural training using RobUST in individuals with chronic cervical (*n* = 5) or high-thoracic (*n* = 1) motor complete SCI, who had previously practiced overground standing with Stand-scES using assistive devices (i.e., a walker). We hypothesized that robotic postural training with Stand-scES would re-enable and/or largely improve upright postural control without any self-assistance provided by the upper limbs.

## Methods

### Participants

Six individuals with chronic cervical or high-thoracic motor complete SCI who were already implanted with a scES unit for the recovery of motor function^6^ participated in this study (Table 1). Prior to enrollment in this study, these individuals had already undergone an average of 112 ± 92 overground stand training sessions with Stand-scES using assistive devices (i.e., a standing apparatus or walker^5^) as part of other interventional studies, and had demonstrated the ability to stand with bilateral independent knees extension.

**Table 1.** Characteristics of the Research Participants

Pub ID	Age (Years)	Sex	Time since injury (Years)	Level of injury	AIS	Time since scES implant (Years)	Stand training (*n*)
A96	29	F	5.3	C4	A	1.7	79
A101	33	M	4.2	C3	A	1.6	160
A82	37	M	8.8	C4	A	1.2	41
B45	36	M	9.3	C7	B	0.3	11
B07	35	M	14.4	T2	B	11.0	265
B23	38	M	9.4	C4	B	6.1	116

Pub ID, publication identifier; level of injury, neurological level of the lesion by AIS (American Spinal Injury Association [ASIA] Impairment Scale); C, cervical; T, thoracic; scES, spinal cord epidural stimulation; stand training: number of stand training sessions with scES and assistive device for self-balance assistance performed prior to the beginning of this study.

The research participants signed an informed consent for scES, activity-based training, physiological monitoring studies, and publication of the related results. This study was conducted according to the standards set by the Declaration of Helsinki and was approved by the University of Louisville Institutional Review Board (IRB #17.1024).

### Spinal cord epidural stimulation implant and parameters

During the scES surgical implantation procedure, a midline bilateral laminotomy was performed typically at the L1-L2 disk space. An electrode array with 16 contacts (Medtronic Specify 5–6-5 lead) was placed into the epidural space at midline. Electrophysiological mapping was performed after initial placement to optimize the location of the paddle electrode based on evoked responses recorded from bilateral surface electromyography (EMG) electrodes (Motion Lab Systems, Baton Rouge, LA) placed over representative lower limb muscles. After the final placement of the electrode array, the electrode lead was tunneled subcutaneously and connected to the neurostimulator (Medtronics, Intellis in participants A96, A101, A82, B45 and B07; RestoreADVANCED in participant B23).

In this study, tonic task-specific Stand-scES was implemented to promote standing. The individual-specific scES parameters applied are reported in Figure S1. All research participants had undergone the process of selection of Stand-scES parameters prior to the beginning of the present study as a result of the enrollment in previous interventional studies that included standing. The approach implemented for the selection of Stand-scES parameters is reported in previous publications by our group.^30–32^ Electrode configuration and stimulation frequency remained constant throughout the present study in five of the six participants (Fig. S1).

### Experimental protocol

Stand-scES was applied to the research participants during all experimental and training sessions of this study. The data herein reported were collected immediately prior to the beginning of robotic postural training (Pre), after 45 ± 7 (Mid), and after 80 ± 10 robotic postural training sessions. At each time point, experimental sessions were devoted to the assessment of (1) steady upright postural control, and (2) proactive upright postural control, in which self-initiated trunk movements and upper limb reaching movements were attempted while standing. Research participants underwent two acclimation sessions prior to data collection at the beginning of the study.

### Robotic Stand Trainer

The RobUST used in this study has been previously described in detail,^29^ and the feasibility of its implementation in a population of individuals with SCI who are unable to stand independently was also assessed.^33^ The device consists of an aluminum frame with 12 motors (Maxon Motor, Switzerland) mounted on it for controlling forces applied by cables to human subjects. The cables are routed from the motors through pulleys and connected to a dedicated harness at the trunk and pelvis. In this study, the harness trunk belt (width: 11 cm) was positioned with its top margin below the axilla of the research participant, and shoulder straps connected to the trunk belt contributed to its tight wearing. The harness pelvic belt was centered on the anterior superior iliac spines and was secured by additional thigh straps. Four cables were attached to each harness belt to apply planar forces; dedicated sensors measured the tension applied at each cable.

**FIG. 1.**
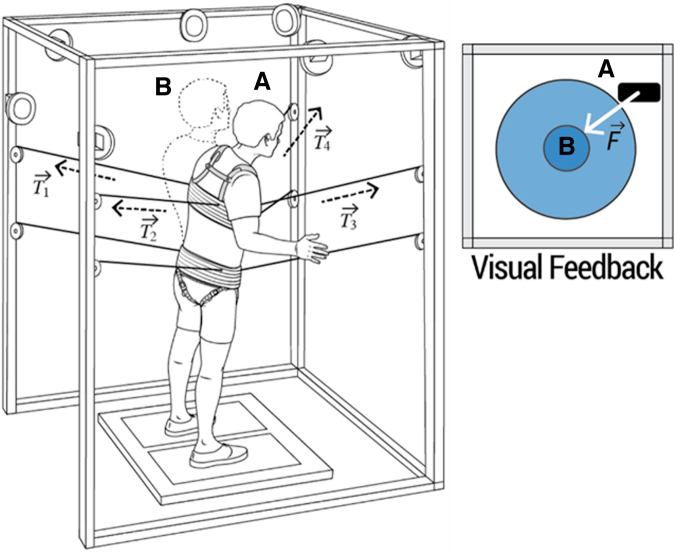
Visual representation of robotic upright stand trainer (RobUST) force field (FF) for trunk assistance as needed. Visual feedback in front of the participant shows the circular FF boundary and the trunk position in real time. When the trunk moves beyond the FF boundary **(A)** the assistive FF is activated, and the restoring force $\vec{F}$, resulting from the application of tension $\vec{T}$ to the four cables, brings the trunk within the boundary and closer to the neutral standing position **(B)**.

The RobUST platform is equipped with eight infrared cameras (Vicon Bonita 10; Denver, CO), which track the motion of the human subject during standing. The motion capture system is used to record the current position of the trunk and pelvic harness belts to calculate the desired forces applied by the device. In this study, RobUST provided assistance as needed at the trunk of research participants. Specifically, a virtual circular boundary (i.e., force field [FF]) is programmed around the individual (Fig. 1), so that they can move freely within the FF boundary, while a restoring force is applied when the trunk moves beyond the FF boundary (e.g., loss of balance control), bringing it back within the boundary and closer to the neutral standing position (Fig. 1; Supplementary Video 1).

In this study, RobUST also applied a constant force of 80 N to the pelvis harness in order to facilitate appropriate hip extension and pelvic tilt, with additional manual trainer's assistance provided as necessary. This constant force level was found optimal for pelvis stability while allowing the pelvis to safely move when excessive forces were generated, such as during unexpected muscle spasms.

### Experimental procedures

Standing upright postural control was assessed overground, within the RobUST frame. Stand-scES was applied while the research participant was seated on their wheelchair near the RobUST frame. Participants used the handlebars in front of them to partially pull themselves while trainers positioned at the knees, pelvis, and trunk manually assisted the sit to stand transition. When the sit to stand transition was completed, if the knees flexed beyond the normal standing posture, external manual assistance by a trainer was provided distal to the patella to promote knee extension. Two trainers were also positioned at the trunk and pelvis to provide additional manual assistance if needed.

Steady and proactive postural assessments were performed without any self-assistance provided by upper limbs (i.e., free-hands) under two conditions: (1) independent trunk control and trainer's manual assistance at the pelvis (Hip-assist), and (2) RobUST assistance as needed at the trunk and constant assistance at the pelvis (RobUST-assist). Real-time visual feedback of the trunk position, cardinal axes, and circular FF boundary was always provided in front of the research participants.

#### Steady upright postural control assessment

The goal of steady upright postural assessment was to stand as steadily as possible for 1 min. Shorter steady standing events resulted from the loss of postural control, requiring the trainer's manual assistance at the trunk and/or the placement of the participant's upper limb(s) on the fixed handlebars in front of them.

#### Proactive upright postural control assessments

Proactive upright postural control was assessed while the research participants attempted self-initiated trunk movements during standing. The goal of antero-posterior (AP) and medio-lateral (ML) trunk movements was to achieve the largest AP or ML peak-to-peak trunk displacement while minimizing the distance traveled away from the cardinal axis (i.e., error). During circular (Circle) trunk movements, participants were instructed to generate a circular movement with the trunk as close as possible to the virtual circular boundary provided by the visual feedback. For each trunk movement, standing condition and time point, three attempts were performed and the one with the largest trunk displacement was considered for further analysis. Attempts needing the trainer's manual assistance at the trunk and/or placement of the participant's upper limb(s) on the handlebars were considered failed attempts, which were not considered for further analysis. 

In four participants (A96, B45, B07, B23), proactive upright postural control was also assessed by implementing self-initiated upper limb reaching movements during hip-assist standing. Starting with the upper limbs hanging vertically down, forward arm reaching movements were attempted with the dominant upper limb (one-arm reaching) or with both upper limbs simultaneously (two-arm reaching). For each arm movement and time point, three attempts were performed, and the one with the largest anterior distance covered was considered for further analysis. Attempts needing the trainer's assistance at the trunk and/or placement of the participant's upper limb(s) on the handlebars were considered failed attempts, which were not considered for further analysis.

#### RobUST Force Field settings for trunk assistance as needed

The FF circular boundary characteristics to assist trunk control during RobUST-assist standing were determined for each participant as follows. Initially, the FF radius was set at 2 cm, and the assistive force magnitude at 15% BW. The individual was asked to initially maintain steady upright posture, and then attempt to perform a self-initiated forward trunk movement to reach the FF boundary displayed on the visual feedback (Fig. 1), and finally to bring the trunk back to a neutral standing position. If the individual was not able to maintain independent trunk control during stable standing, FF radius remained at 2 cm and different FF magnitudes (up to 35% BW) were tested to optimize trunk assistance. If the individual was able to perform and control the forward trunk movement, FF radius was progressively increased by 1 cm (up to a maximum of 6 cm) until the task could be successfully performed. Different FF magnitudes (up to 35% BW) were subsequently tested to optimize trunk assistance.

### Data acquisition

Motor force, kinematic data, force platforms data, and EMG of the following trunk and lower limb muscles were collected in this study. A LabVIEW PXI system (National Instruments, Austin, TX) and load cells (LSB302 Futek, CA) were used to record force generated by the DC motors (Maxon Motor, Switzerland) at 200 Hz. Vicon Nexus motion capture system (Vicon, Denver, CO) was used to record kinematics sampled at 100 Hz, via eight Vicon Bonita infrared cameras (Vicon, Denver, CO). Retro reflective markers were placed at the shoulders, trunk, and pelvis, and their position was tracked in real time to control the forces applied by RobUST. During the device calibration process, reflective markers were also placed on the arms, pulleys, and cable-harness attachments. Two force platforms were used to measure ground reaction forces, which were collected in the Vicon Nexus system at 1000 Hz. EMG activity of the right (R) and left (L) upper trapezius (UT), adductor (AD), vastus lateralis (VL), medial hamstring (MH), anterior (TA), and medial gastrocnemius (MG) was recorded by bipolar surface electrodes with a fixed inter-electrode distance of 2000 Hz using a custom-written acquisition software (National Instruments, Austin, TX).

### Data analysis

A low-pass digital filter was applied to motor and force plate data with a cutoff frequency of 10 Hz.^34^ Kinematic data of the trunk geometrical center to estimate trunk displacement^29^ were low-pass filtered (6 Hz) in Vicon Nexus. Also, a band-pass digital filter (10–500 Hz) was applied to EMG data.

#### Steady upright postural control

The primary outcome for this postural task was the duration that research participants were able to maintain steady postural control. Additionally, mean trunk velocity was calculated as the total distance traveled in the transverse plane by the trunk geometrical center divided by the attempt duration. The variability of vertical ground reaction forces (Fz variability) was assessed by calculating the coefficient of variation (standard deviation/mean) over the steady standing duration. Median frequency and median frequency standard deviation for lower limb EMG activity (average value among the investigated lower limb muscles) were calculated over the steady standing event by Continuous Wavelet Transform as previously detailed.^35^ Integrated EMG (iEMG) normalized by the attempt duration was also calculated for the investigated muscles (UT and lower limb muscles).

#### Proactive upright postural control

The primary outcome to assess upright proactive postural control during self-initiated trunk movements was the distance covered with the trunk in the AP or ML directions, and the area covered with the trunk during circular movements. Trunk displacement for the linear trunk movements (AP and ML) was calculated as the AP or ML peak-to-peak distance covered by the trunk geometrical center. For RobUST-assist attempts, the distance of trunk geometrical center traveled beyond the FF boundary, which resulted from the loss of trunk control, was excluded from displacement calculation. For the circular movements, the area of the circle created by the trunk geometrical center was calculated. For RobUST-assist attempts, only the area covered within the circular boundary was considered for performance calculation.

The trunk movement precision was characterized by the movement error. For AP movements, error was calculated as the average ML point-to-point distance from the cardinal axis to the trunk geometrical center over a given attempt. For ML movements, error was calculated as the average AP point-to-point distance from the cardinal axis to the trunk geometrical center over a given attempt. For the circular movements, error was calculated as the average point-to-point distance from the closest circular boundary point to the trunk geometrical center over a given attempt. iEMG normalized by the attempt duration was calculated for the investigated muscles (UT and lower limb muscles) and expressed as percent of the respective iEMG values assessed during steady standing (baseline). Quantitative information about the coordination pattern between representative antagonist muscles crossing the ankle joint (MG vs. TA) has been obtained based on the approach reported by Rejc and colleagues.^36^ Briefly, each data point of the joint probability density distribution^37^ represents the amplitude relationship of the EMG signals from the two muscles at a given time point. Ten percent of the largest amplitude detected during the self-initiated trunk movement attempts was set as a threshold to define four areas of the plot representing negligible activation of both muscles, the isolated activation of either muscle, or co-contraction (i.e., concurrent activation of both muscles with an amplitude >10% threshold). The data points related to negligible activation of both muscles were discarded, and the number of data points distributed in each of the three remaining areas was finally expressed as a percentage of the data points collected during a given attempt.

The primary outcome to assess upright proactive postural control during forward arm reaching movements was the distance covered by the participant's wrist. Wrist displacement was calculated as the anterior peak distance covered by the wrist from its starting position (upper limb hanging vertically down). When assessing one-arm reaching, the anterior peak distance of the dominant upper limb was considered for analysis. As for two-arm reaching, the average anterior peak distance of both upper limbs was calculated and considered for analysis.

### Robotic postural training

Research participants underwent on average 80 ± 10 training sessions (1 h/day; 5 days/week). Robotic upright postural training was always performed with Stand-scES in the RobUST frame. Stand-scES was applied while the research participant was seated on their wheelchair near the RobUST frame. Participants used the handlebars in front of them to partially pull themselves while trainers positioned at the knees, pelvis, and trunk manually assisted the sit to stand transition. When the sit to stand transition was completed, if the knees flexed beyond the normal standing posture, manual assistance by a trainer was provided distal to the patella to promote knee extension. Two trainers positioned at the trunk and pelvis provided additional manual assistance if needed.

Robotic postural training was performed with free hands (i.e., handlebars or other fixed surfaces were not used for self-balance assistance); each training session consisted of periods of steady standing, self-initiated trunk and arm movements, and trunk perturbations delivered toward the four cardinal directions by RobUST.^28,29^ Points of training progression included the modulation of FF for trunk assistance as needed (i.e., decreased FF magnitude, increased FF radius, FF removed), the increase of self-initiated arm movements complexity with respect to upright postural control (i.e., from lateral arm abduction to one-arm forward reaching to two-arm forward reaching), and the increase of trunk perturbation magnitude. Manual assistance at the pelvis was also gradually implemented to substitute RobUST constant assistive force. Additionally, training progression included the increased amount of dynamic tasks compensated for by a decrease of steady standing time within a session. Seated resting periods occurred when requested by the individuals.

### Statistical analysis

Statistical analysis was performed using JASP (Version 0.18, Netherlands) and GraphPad Prism (version 5.01, California, USA) software. Results are reported as mean and standard deviation. Normal distribution of the data was tested using the Kolmogorov–Smirnov test. The effect of robotic postural training (Pre, Mid, Post) on the outcomes considered in this study was analyzed within each motor task (steady standing, self-initiated trunk movements, arm reaching movements) and standing condition (Hip-assist or RobUST-assist) with one-way repeated measures analysis of variance (ANOVA). Sphericity was verified by Mauchly's test. When the assumption of sphericity was not met, the significance of the F-ratios was adjusted according to the Greenhouse–Geisser procedure. When significant differences were found, a Bonferroni post hoc test was used to determine the exact location of the difference. In cases of violation of the assumption of normality, the Friedman test and subsequent Conover's post hoc tests were implemented. All tests were two sided and the significance level was set as *p* < 0.05. Multiple imputation was implemented to handle the missing secondary outcomes (e.g., EMG, and force plate data describing motor control) when individuals were not able to perform the requested postural task in the hip-assist condition, scoring 0 for the primary outcomes. Multiple imputation was implemented by full conditional metropolis sampler with auto-derived conditional distributions, constrained to minimum and maximum values present in the data set, using the primary outcome variable in the model as predictor.^38^ Blimp Studio (Version 3.2.1) software was used to perform multiple imputation. The magnitude of robotic postural training-promoted improvements (Post vs. Pre) of primary postural control outcomes (steady standing duration, trunk displacement during self-initiated trunk movements, reaching distance during self-initiated arm movements) was also assessed by Cohen's effect size.^39^ Differences resulting in large (0.80–1.29) and very large (≥ 1.30) effect size are highlighted in the text.

## Results

### Steady upright postural control

**FIG. 2.**
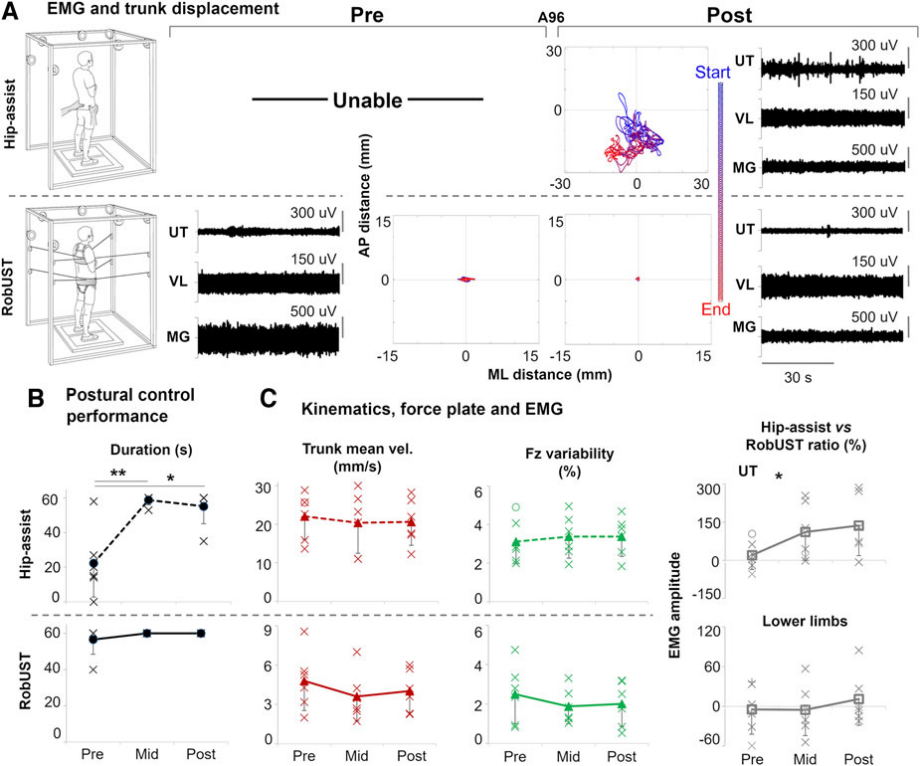
Steady upright postural control. (**A**) Representative trunk displacement and electromyography (EMG) for the upper trapezius (UT), vastus lateralis (VL), and medial gastrocnemius (MG) from participant A96 during stable standing attempts performed with free hands when external manual assistance was provided only at the pelvis (hip-assist, top) and with robotic upright stand trainer (RobUST) assistance (bottom) at Pre and Post training. At Pre, the participant was able to perform this motor task with RobUST assistance while being unable to achieve it during hip-assist standing. Robotic postural training resulted in the participant's ability to perform and complete the motor task at Post training in the hip-assist standing condition. (**B**) Individual (crosses) and mean (black circles) data points (*n* = 6 participants) of the primary outcome of postural control performance (steady standing duration) during hip-assist (top) or RobUST-assist (bottom) at Pre, Mid and Post training. (**C**) Individual data points (crosses, or circle for the imputed data [*n* = 1]) and mean data of kinematic, kinetic, and EMG variables collected at Pre, Mid and Post training. Fz: vertical ground reaction force. Error bars represent standard deviation across subjects. *p < 0.05; **p < 0.01.

Research participants achieved steady standing demonstrating overall continuous lower limb activation patterns (Fig. 2A) that resulted in independent bilateral lower limb extension for the entire attempt duration in four individuals at Pre, and in five individuals at Mid and Post (Table S1). Prior to robotic postural training, five of the six research participants receiving Stand-scES were able to achieve steady standing posture with free hands when manual assistance was provided at the pelvis by a trainer (hip-assist; Fig. 2A and B). However, none of the participants were able to maintain steady standing for the entire 1-min task. Conversely, RobUST assistance as needed at the trunk and constant assistance at the pelvis immediately facilitated steady standing with free hands in all participants, with five of them successfully completing the 1-min task (Fig. 2A and B). Robotic postural training promoted a very large and significant improvement in steady postural control performance during hip-assist standing at Mid training (Effect Size [ES] = 2.61; *p* = 0.009) and a similar trend at Post training (ES = 2.11; *p* = 0.027), as indicated by the increased standing event duration that approached the 1-min target (Fig. 2B). This postural control improvement was not associated with changes in trunk mean velocity (*p* = 0.633) or other features that are relevant for stable standing in this population such as ground reaction force variability (*p* = 0.787; Fig. 2C), lower limb EMG median frequency, and its standard deviation (*p* = 0.849 and *p* = 0.605, respectively, Fig. S2). Compared with RobUST-assist, postural training had a significant effect (*p* = 0.042) on UT activation during hip-assist standing (Fig 2C), which tended to increase from Pre to Mid (*p* = 0.167) and Post (*p* = 0.117).

### Proactive upright postural control – self-initiated trunk movements

**FIG. 3.**
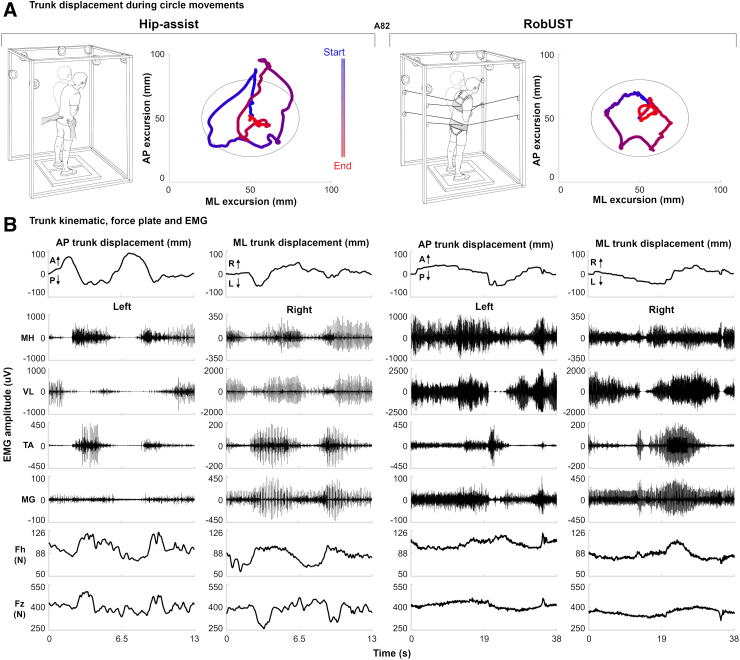
Exemplary self-initiated trunk circular movements. (**A**) Representative trunk displacement plotted from start (blue) to end (red) of the trial (AP: antero-posterior; ML: medio-lateral). (**B**) Trunk kinematics (A: anterior; P: posterior; R: right; L: left), electromyography (EMG) activity of lower limb muscles (MH: medial hamstrings; VL: vastus lateralis; TA: tibialis anterior; MG medial gastrocnemius) and ground reaction forces (Fz: vertical force; Fh: horizontal resultant force) from participant A96 during a Post training circular self-initiated trunk movement attempt during hip-assist standing (left) and with robotic upright stand trainer (RobUST) assistance (right). Note the EMG activation pattern modulation associated with changes in trunk and ground reaction forces.

**FIG. 4.**
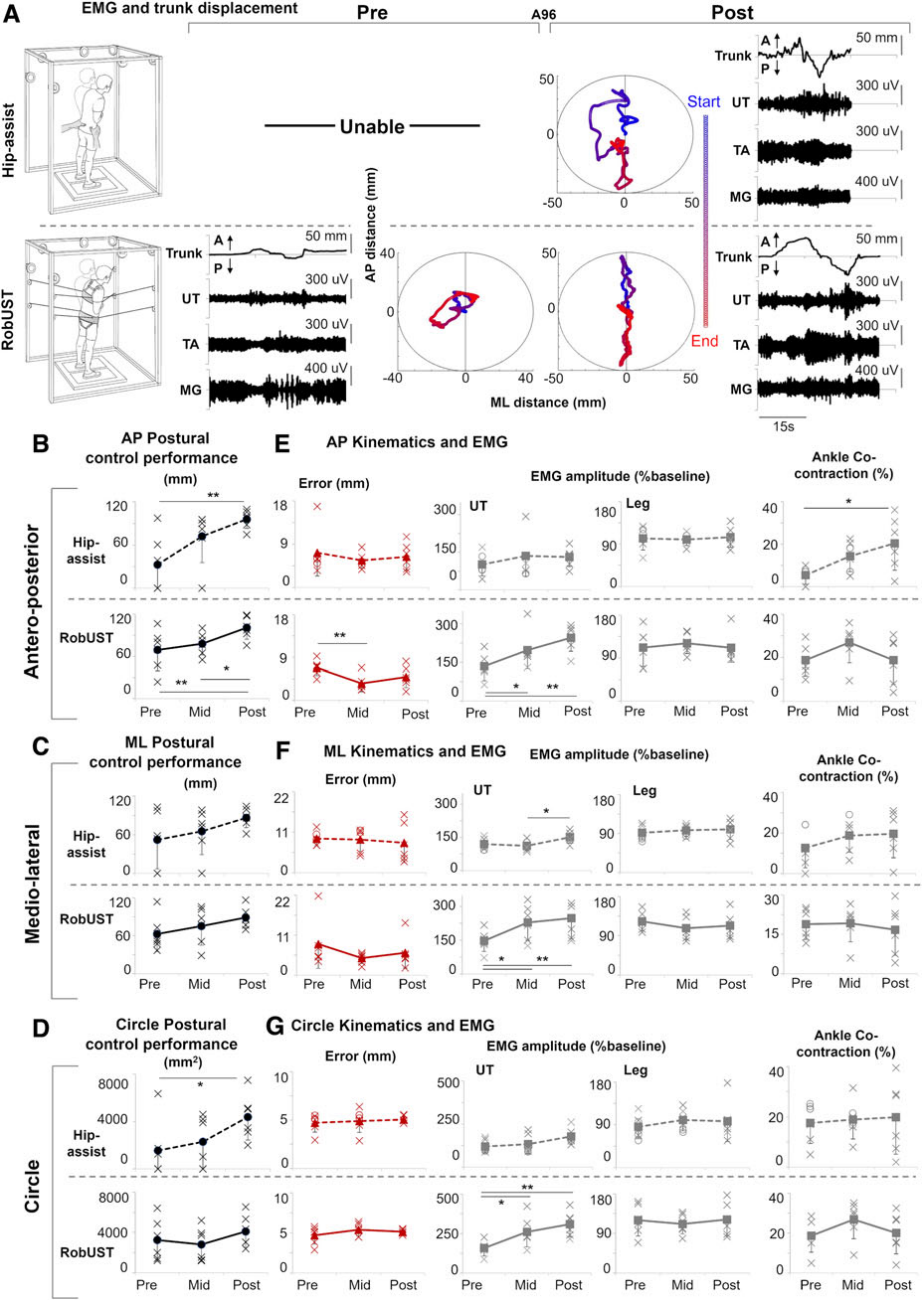
Proactive upright postural control by self-initiated trunk movements. (**A**) Representative trunk displacement plotted from start (blue) to end (red) of the trial (AP: antero-posterior; ML: medio-lateral) and electromyography (EMG) for the upper trapezius (UT), tibialis anterior (TA), and medial gastrocnemius (MG) from participant A96 during a self-initiated, AP trunk movement attempt performed with free hands when external manual assistance was provided only at the pelvis (hip-assist standing, top) and with robotic upright stand trainer (RobUST) assistance (bottom) at Pre and Post training. At Pre training, the participant was able to attempt the motor task with RobUST assistance while being unable to attempt during hip-assist standing. Robotic postural training resulted in the participant being able to complete the motor task at Post training during hip-assist standing and improve performance with RobUST assistance. (**B**–**D**) Individual (crosses) and mean (black circles) data (*n* = 6) of the primary outcome of postural control performance (trunk displacement) during AP (**B**), ML (**C**), and circular (Circle; **D**) trunk movement tasks performed while hip-assist standing or with RobUST assistance at Pre, Mid, and Post training. Generally, robotic postural training led to increases in performance. (**E**–**G**). Individual data points (crosses, or circles for imputed data) and mean data of kinematic, kinetic, and EMG variables collected with RobUST-assist or hip-assist standing while performing AP (**E**), ML (**F**), and Circle (**G**) trunk movements. Error bars represent standard deviation (SD) across subjects. *p < 0.05; **p < 0.01.

Proactive upright postural control was evaluated while the SCI participants receiving Stand-scES attempted self-initiated trunk movements during RobUST-assist and hip-assist standing (Fig. 3A). AP, ML, and Circle trunk movements were attempted with the goal of covering the largest distance in the cardinal directions (AP or ML) or the largest circular area (Circle) while real-time visual feedback of trunk position was provided. These motor tasks resulted in trunk displacement and ground reaction forces modulation associated with relevant lower limb EMG responses (Figs. 3B and 4A).

Prior to robotic postural training, only three (AP and Circle) or four (ML) of the six participants were able to perform these motor tasks during hip-assist standing (Fig. 4A–D). RobUST assistance immediately enabled all participants to perform these self-initiated trunk movements while standing with free hands. After robotic postural training, all research participants regained the ability to control the self-initiated trunk movements while standing with hip-assist (Fig. 4A–D). Robotic postural training promoted very large and significant improvements in proactive upright postural control during hip-assist standing as assessed by the increased AP (ES = 2.10; *p* = 0.003) and Circle (ES = 1.27; *p* = 0.010) trunk movement performance. A similar large trend (ES = 0.89; *p* = 0.073) was observed for ML trunk movement performance. The precision (i.e., error) of these movements was not significantly affected by training. RobUST-assist proactive postural control showed similar training-induced positive trends, with significant and very large (ES = 1.34) performance improvements found for AP movements (Fig. 4B). For this standing condition and motor task, trunk movement precision significantly improved at Mid-training as indicated by the smaller error detected (*p* = 0.008). Similar trends were also observed during RobUST-assist ML trunk movements (Fig. 4F). Independent bilateral lower limb extension was achieved in all successful attempts considered for analysis, except for research participant B07 (Table S2).

Different mechanisms appeared to be associated with the improved proactive postural control demonstrated in the two standing environments. Significant (*p* = 0.019) training-induced increase in ankle co-contraction was demonstrated during AP trunk movements in hip-assist standing (Fig. 4E), and the same trend was also present during ML (*p* = 0.071; Fig. 4F). Conversely, significant training-induced increase in UT activation was found during AP (*p* = 0.003), ML (*p* = 0.009), and Circle (*p* = 0.004) trunk movements generated while standing with RobUST; similar statistically significant trends were already observed at Mid training (Fig. 4E–G).

### Proactive upright postural control – arm reaching

**FIG. 5.**
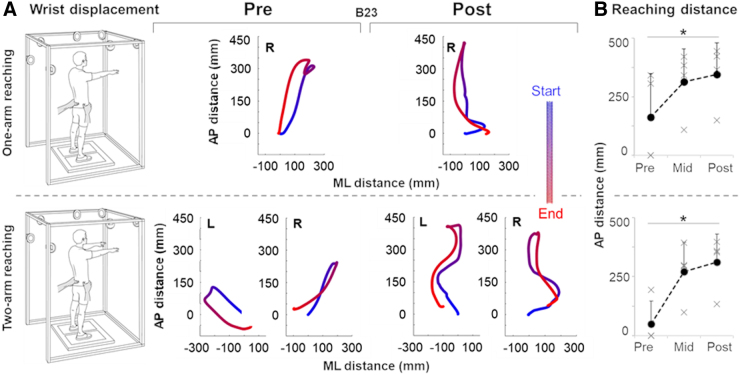
Proactive upright postural control by arm reaching. (**A**) Representative right (R) and left (L) wrist excursion from participant B23 during a one-arm (top) and two-arm forward reaching task, plotted from the start (blue) to the end (red) of the trial, while standing with free hands when external manual assistance was provided only at the pelvis (hip-assist standing) at Pre and Post training. (**B**) Individual (crosses) and mean (black circles) data of peak anterior reaching distance during one-arm (top) and two-arm (bottom) forward reaching while standing with free hands (*n* = 4). Error bars represent standard deviation (SD) across subjects. *p < 0.05.

Proactive upright postural control was also evaluated in a subgroup (*n* = 4) of participants while attempting self-initiated, forward arm reaching movements during hip-assist standing with Stand-scES (Fig. 5A). Prior to robotic postural training, only two participants were able to perform one-arm reaching movements, and only one participant successfully performed forward arm reaching with both upper limbs simultaneously (Fig. 5B). After training, all four individuals were able to successfully perform both reaching tasks while maintaining standing postural control. Robotic postural training promoted a large (ES = 1.12), significant (*p* = 0.048) increase in one-arm reaching distance, and a very large (ES = 2.68) and significant (*p* = 0.030) increase in two-arm reaching distance (Fig. 5B). Similar, non-significant postural control improvements were already demonstrated at Mid training. During all successful attempts considered for analysis, three of the four participants always maintained independent bilateral lower limb extension (Table S2).

## Discussion

Robotic postural training with Stand-scES re-enabled and/or largely improved fundamental aspects of standing postural control in individuals with chronic cervical or high-thoracic motor complete SCI that had previously undergone stand training with Stand-scES using a walker or a standing frame for self-balance assistance. After robotic postural training, participants were able to maintain steady standing and generate self-initiated trunk and upper limb movements without any self-balance assistance provided by upper limbs while receiving only external assistance for pelvic control. These relevant functional improvements were associated with training-induced activation pattern adaptations observed above (UT muscle) and below (lower limb muscles) the level of injury.

In the last decade, our group and others showed that activity-based training with individual-specific Stand-scES can promote the recovery of standing overground with independent lower limbs extension in individuals with chronic, motor complete SCI self-assisting postural control with their upper limbs on assistive devices.^5–7,10,31,35^ Five of the six participants of the present study consistently demonstrated independent bilateral lower limbs extension during the upright postural control tasks performed with free hands (Tables S1 and S2). Tonic Stand-scES parameters are configured with the goal of enabling the lumbosacral spinal circuitry to use peripheral sensory information, and possibly residual supraspinal inputs, as sources of motor control, rather than directly driving motor pattern generation.^6,8,31,40,41^ In particular, lower limb loading and extension associated with the sit-to-stand transition can be interpreted by the spinal circuitry to modulate negligible activation (in sitting) into robust, overall continuous EMG patterns effective for standing, without any change in stimulation parameters.^31,35^

In the present study, we have implemented RobUST with the goal of providing an enriched ensemble of sensory information for bipedal upright postural control. RobUST immediately re-enabled the practice of steady and dynamic standing with free hands in a population of cervical or high-thoracic motor complete SCI (Figs. 2–4). This is relevant because providing mechanical self-stabilization with upper limbs during standing can lead to altered postural control strategies that reduce or even suppress lower limb neuromuscular responses while improving overall stability because of upper limb contribution.^11,12,42–44^ We have also recently investigated the effects of upper limb self-stabilization on postural responses to trunk perturbations during standing with Stand-scES.^28^ These findings supported the view that a free-hands condition can promote lower limb responses that are generally more frequent, larger in magnitude, and overall more appropriately modulated than standing with self-balance assistance, even in a motor complete SCI population receiving spinal cord neuromodulation.

In the present study, self-initiated trunk movements during both RobUST-assist and hip-assist standing led to controlled trunk displacement and modulation of vertical and horizontal ground reaction forces resulting in relevant lower limb EMG pattern modulation (Fig. 3). These results are consistent with the interpretation that the human spinal cord below the level of a motor complete SCI can generate meaningful postural responses when tonic lumbosacral scES is applied with the goal of modulating the excitability of the spinal circuitry. Similar perspectives were previously supported by animal studies implementing scES, suggesting that the spinal postural circuitry substantially contributes to postural control, and that this circuitry is not functional after SCI primarily because of the loss of tonic supraspinal drive.^25,45,46^ Our current results also support the view that somatosensory information plays a major role in the generation and control of postural responses,^47–49^ and that limb loading modulation is an important component of postural responses.^14,20,28^

Robotic postural training promoted large and significant improvements of upright postural control as indicated by the regained and/or increased ability to maintain steady standing (Fig. 2A and B) and control larger self-initiated trunk movements (Fig. 4A–D) and arm reaching movements (Fig. 5). Different training-induced postural control adaptations involving both the upper trunk and lower limb muscles were observed. For example, indexes of increased UT activation were associated with training-induced improvements of steady postural control during hip-assist standing (Fig. 2C) and during self-initiated trunk movements with RobUST-assist (Fig. 4E–G). All participants were able to voluntarily activate UTs to control shoulder and head movements. It is plausible that information about head orientation from visual and vestibular systems was interpreted to generate postural responses by activating the upper trunk muscles above the level of injury, and that this postural strategy was reinforced with training to control steady hip-assist standing and self-initiated trunk movements with RobUST-assist.^46,50^

Evidence from decerebrated animal models suggests that key components of the nervous mechanisms responsible for supraspinal postural control reside in the brainstem and cerebellum.^46^ Further, visual and vestibular information appear to play a major role in the supraspinal postural control mechanisms predominantly devoted to phasic corrective commands, which are sent to the spinal cord via vestibulospinal, reticulospinal, rubrospinal, and corticospinal descending pathways. On the other hand, training-promoted improvements in self-initiated trunk movements during hip-assist standing were associated with trends of increased co-contraction of antagonist ankle muscles (TA and MG; Fig. 4E–G). Increased co-contraction of antagonist leg muscles can be interpreted as a mechanism to increase joint stiffness and overall stability, which is also observed in able-bodied populations with aging and disuse while performing different motor tasks.^51–54^ The repetitive practice of a motor task can promote use-dependent strengthening of the targeted sensorimotor pathways to result in behavioral, neurochemical, and physiological adaptations.^55^ Training-induced neural adaptations may have promoted the remodeling of synaptic connections among spinal inhibitory and excitatory interneurons projecting to motorneurons, and/or adaptations related to afferent synaptic inputs and these spinal interneurons.^56^ The reorganization of other mechanisms of inhibitory control that are typically impacted by SCI (i.e., non-reciprocal Ib inhibition; reciprocal inhibition) may have also occurred.^56–58^ It is worth mentioning that the assistance-as-needed paradigm provided at the trunk by RobUST conceivably contributed to the observed motor relearning by promoting intrinsic variability that is important for the spinal circuitry controlling the execution of a motor task after SCI.^59,60^

Although the key goal of this study was to determine the combined effect of scES and RobUST postural training on upright steady and proactive postural control, the independent effects of RobUST postural training and scES alone on standing postural control need to be specifically evaluated in future studies. However, it is worth mentioning that individuals with a chronic, motor complete SCI are unlikely to demonstrate relevant motor recovery by activity-based recovery training alone, even if it is intense.^6,9,61,62^ Also, the participants had undergone substantial stand training with Stand-scES using a standing frame or walker for self-balance assistance prior to this study (Table 1), and they underwent two acclimation sessions with the RobUST prior to pre-training data collection. Therefore, even though a control group is lacking at this stage, the findings of this study support the perspective that, even after a motor complete SCI, the human spinal cord can re-learn aspects of standing postural control if appropriate training and neuromodulation are provided. Also, a larger cohort of participants is needed to assess to what extent the relevant postural control improvements found in this study can be expected in a broader SCI population.

## Conclusion

In summary, scES combined with free-hands robotic postural training promoted significant and large upright postural control improvements in a small group of individuals with chronic cervical or high-thoracic motor complete SCI who had previously practiced standing overground with scES using assistive devices for self-balance assistance. The observed postural control improvements were related to a variety of activation pattern adaptations both below and above the level of injury. From a mechanistic standpoint, the findings herein reported suggest that the human lumbosacral spinal cord below the level of injury can generate meaningful postural responses when its excitability is modulated by scES and can be trained to improve these responses in conjunction with activation patterns above the level of injury. Future studies should confirm whether the repetitive practice of postural control tasks with (1) free hands and (2) robotic assistance as needed are the two key determinants of the superior postural control re-learning observed here. From a functional perspective, the improvements in upright postural control can increase the potential for the individual to interact with the environment while standing. With the implementation of scES in the home and community environment at the horizon for the SCI population, the observed functional gains might further support the safe and effective practice of standing, aided by an assistive device when needed, while also enabling trunk and upper limb reaching movements. Finally, future studies are warranted to assess whether the observed postural control gains may translate to improvements in the control of other motor tasks such as walking and sitting, and whether robotic postural training concurrently targeting trunk and pelvic control may promote further postural control improvements.

## Supplementary Material

Supplemental data

Supplemental data

Supplemental data

Supplemental data

Supplemental data

## Data Availability

Data that support the findings herein reported will be made available through material transfer agreement upon reasonable request.

## Transparency, Rigor, and Reproducibility Summary

This human interventional pilot study, which involved six individuals with motor complete spinal cord injury were already implanted with a spinal cord epidural stimulation unit for the recovery of motor control, was not formally registered. The analysis plan was not formally pre-registered. A sample size of six participants was planned based on the available grant support, and on previous proof-of-principle studies that were successful in investigating mechanisms of human motor control with neuromodulation after spinal cord injury. Participants and investigators could not be blinded during data collection, and investigators were aware of relevant characteristics of the assessed motor tasks during data analysis. Biomechanical data were analyzed as they were collected. The RobUST was custom-built by the investigators; schematics will be made available upon request. Key inclusion criteria and outcome evaluations are established standards. There are no current or planned replication studies ongoing to our knowledge. All materials used to conduct the study were obtained by the investigators; de-identified data from this study and analytic code used for data analysis are not available in a public archive and will be made available through material transfer agreement upon reasonable request. This article will be published with Open Access license.

## Abbreviations Used

AD: adductor
ANOVA: analysis of variance
AP: antero-posterior
BW: body weight
EMG: electromyography
FF: force field
MG: medial gastrocnemius
MH: medial hamstring
ML: medio-lateral
RobUST: robotic upright stand trainer
SCI: spinal cord injury
ScES: spinal cord epidural stimulation
TA: tibialis anterior
UT: upper trapezius
VL: vastus lateralis
