# Supplementary figures and images for: Robotic Postural Training With Epidural Stimulation for the Recovery of Upright Postural Control in Individuals With Motor Complete Spinal Cord Injury: A Pilot Study
Source: Neurotrauma Rep. 2024 Mar 15;5(1):277–92. doi: 10.1089/neur.2024.0013 (PMC10956531; doi:10.1089/neur.2024.0013)

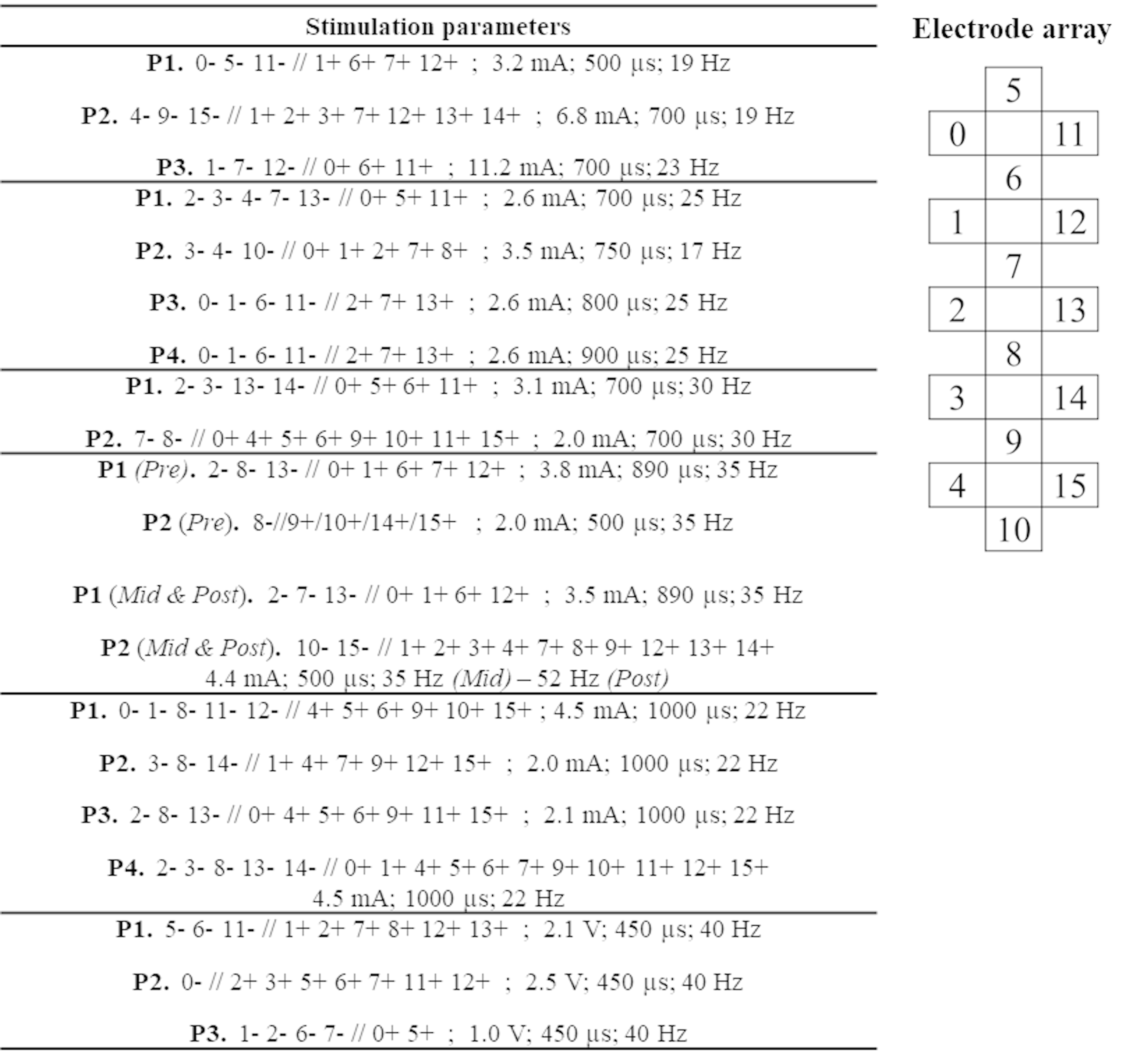

Supplement: Supplemental data [file Suppl_FigureS1.tif]

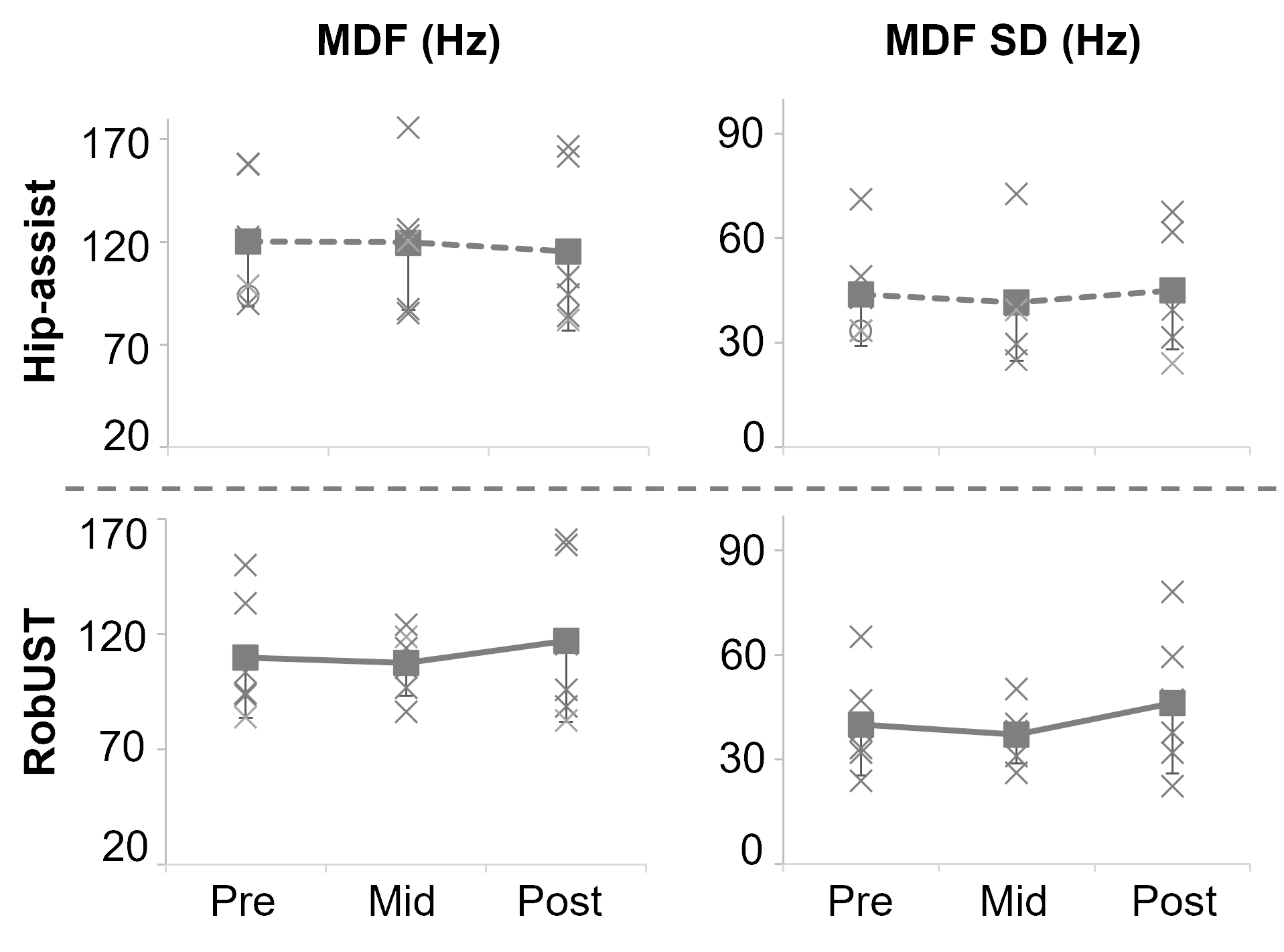

Supplement: Supplemental data [file Suppl_FigureS2.tif]
