# Supplementary material for: Robotic Postural Training With Epidural Stimulation for the Recovery of Upright Postural Control in Individuals With Motor Complete Spinal Cord Injury: A Pilot Study
Source: Neurotrauma Rep. 2024 Mar 15;5(1):277–92. doi: 10.1089/neur.2024.0013 (PMC10956531; doi:10.1089/neur.2024.0013)
Supplement: Supplemental data [file Suppl_TableS1.docx]

**Supplemental Table 1.** Amount of independent bilateral lower limb extension, expressed as percentage of total attempt duration, during steady upright postural control assessed at Pre, Mid and Post robotic postural training. Pub ID: publication identifier.

| **Pub ID** |  | **Hip-assist**  (% duration) | | |  | **RobUST**  (% duration) | | |
| --- | --- | --- | --- | --- | --- | --- | --- | --- |
|  |  | **Pre** | **Mid** | **Post** |  | **Pre** | **Mid** | **Post** |
| A96 |  | 100 | 100 | 100 |  | 100 | 100 | 100 |
| A101 |  | 0 | 100 | 100 |  | 70 | 100 | 100 |
| A82 |  | 100 | 100 | 100 |  | 100 | 100 | 100 |
| B45 |  | 100 | 100 | 100 |  | 100 | 100 | 100 |
| B07 |  | 0 | 0 | 0 |  | 0 | 0 | 70 |
| B23 |  | 100 | 100 | 100 |  | 100 | 100 | 100 |
