# Supplementary material for: Robotic Postural Training With Epidural Stimulation for the Recovery of Upright Postural Control in Individuals With Motor Complete Spinal Cord Injury: A Pilot Study
Source: Neurotrauma Rep. 2024 Mar 15;5(1):277–92. doi: 10.1089/neur.2024.0013 (PMC10956531; doi:10.1089/neur.2024.0013)
Supplement: Supplemental data [file Suppl_TableS2.docx]

**Supplemental Table 2.** Amount of independent bilateral lower limb extension, expressed as percentage of total attempt duration, during proactive upright postural control assessed by self-initiated trunk and arm movements at Pre, Mid and Post robotic postural training. For each time point, the number of motor tasks that the participants were able to successfully generate, which are considered for analysis, are reported within parenthesis. Trunk movement tasks were: antero-posterior, medio-lateral and circular movements. Arm reaching tasks were: one-arm and two-arm reaching. Pub ID: publication identifier. *N/A*: not applicable (motor task not tested).

| **Pub ID** |  | **Trunk movements Hip-assist**  % duration (*tasks n.*) | | |  | **Trunk movements RobUST**  % duration (*tasks n.*) | | |  | **Arm reaching Hip-assist**  % duration (*tasks n.*) | | |
| --- | --- | --- | --- | --- | --- | --- | --- | --- | --- | --- | --- | --- |
|  |  | **Pre** | **Mid** | **Post** |  | **Pre** | **Mid** | **Post** |  | **Pre** | **Mid** | **Post** |
| A96 |  | - (*0*) | 100 (*1*) | 100 (*3*) |  | 100 (*3*) | 100 (*3*) | 100 (*3*) |  | - (*0*) | 100 (*2*) | 100 (*2*) |
| A101 |  | - (*0*) | 100 (*1*) | 90 (*3*) |  | 27 (*3*) | 100 (*3*) | 100 (*3*) |  | *N/A* | *N/A* | *N/A* |
| A82 |  | 100 (*3*) | 100 (*3*) | 100 (*3*) |  | 100 (*3*) | 100 (*3*) | 100 (*3*) |  | *N/A* | *N/A* | *N/A* |
| B45 |  | 100 (*3*) | 100 (*3*) | 100 (*3*) |  | 100 (*3*) | 100 (*3*) | 100 (*3*) |  | - (*0*) | 100 (*2*) | 100 (*2*) |
| B07 |  | 0 (*2*) | 0 (*3*) | 0 (*3*) |  | 0 (*3*) | 0 (*3*) | 0 (*3*) |  | 0 (*1*) | 0 (*2*) | 0 (*2*) |
| B23 |  | 100 (*3*) | 100 (*3*) | 100 (*3*) |  | 100 (*3*) | 100 (*3*) | 100 (*3*) |  | 100 (*2*) | 100 (*2*) | 100 (*2*) |
